# Prevalence of Anaemia and Evaluation of Transferrin Receptor (sTfR) in the Diagnosis of Iron Deficiency in the Hospitalized Elderly Patients: Anaemia Clinical Studies in Chile

**DOI:** 10.1155/2012/646201

**Published:** 2012-05-03

**Authors:** Mauricio López-Sierra, Susana Calderón, Jorge Gómez, Lilian Pilleux

**Affiliations:** ^1^Hematology Unit, Institute of Medicine, Faculty of Medicine, Universidad Austral de Chile, Bueras 1003 CP 5090000 Valdivia, Chile; ^2^Institute of Public Health, Faculty of Medicine, Universidad Austral de Chile, Valdivia, Chile

## Abstract

Iron constitutes the most prevalent nutritional deficiency worldwide. In Chile, anaemia epidemiological data is scarce, evaluating mainly children and women. Our objective was to determine prevalence of anaemia in an inpatient elderly population (≥60 years) and assess the usefulness of sTfR levels analyzed by other authors as a good predictor in the differential diagnosis of iron deficiency anaemia and anaemia of chronic disease. *Method.* We studied medical patients admitted at Hospital of Valdivia (HV), Chile, in a 2month period. World Health Organization criteria were used for anaemia. *Results.* 391 patients were hospitalized, average age 62.5 years, 247 elderly and 99 of which had anaemia. Anaemia was normocytic in 88.8%, and we observed: low serum iron in 46.3%, low ferritin 10.1%, high TIBC 2%, low % transferrin saturation (Tsat) 40%, and high sTfR 25%. *Conclusions.* As a first figure known in Chile, the prevalence of anaemia in the elderly inpatient was 40.1%. Our findings encourage us to promote the implementation of sTfR determination in the clinical setting to analyze the state of erythropoiesis in patients with chronic diseases wich commonly occurs in elderly.

## 1. Introduction

Iron (Fe) is an essential metal ion for living beings; although it is the fourth most abundant mineral in the earth's crust, it is the most prevalent nutritional deficiency worldwide [1]. It participates in a variety of vital physiological processes such as oxygen transportation, energy production in the brain by cytochrome oxidase, enzymatic cofactor in the synthesis of neurotransmitters and myelin [2, 3]. The main consequence of iron deficiency is the generation of anaemia which allows us to estimate its prevalence in a given population indirectly by red blood cell counting. However, this approach has the limitation of including other aetiologies. The worldwide prevalence of iron deficiency is approximately 30%, resulting in close to 2 billion people with anaemia of this cause. In developing countries, the prevalence of anaemia among pregnant women and children under two years exceeds 50% [4, 5]. In Chile, the epidemiological anaemia data is scarce and sectored, with values ranging from 5.1% in women [6] up to 36% in infants of low socioeconomic status [7].

The impact of iron deficiency occurs not only in the hematopoietic system and is more evident in the early stages of life affecting preschool children who suffer from behavioral and affective disorders [8], increased infection susceptibility, and pregnant women having increased risk of preterm delivery, low birth weight, and death in the newborn (NB). Children, especially premature NB children from mothers with iron deficiency, adolescent girls, and women of childbearing age [9, 10] represent the most vulnerable population for this deficiency because of their increased demand and/or physiological loss of Fe.

There is no epidemiological data available of iron deficiency in the elderly, but they are expected to have a higher prevalence of anaemia than in the general population, since longevity is associated with a variety of physiological dysfunctions, chronic and inflammatory diseases, and occasionally inadequate diet that lower reserves and availability of Fe. Clinical manifestations of anaemia in the elderly add to changes in sensory organs, increasing the risk of falls, with a decline in mobility and loss of autonomy [11] that results in an increase in health expenses.

When facing a patient with iron deficiency anaemia (IDA), the hematimetric and ferrokinetic classical standards can be altered by concomitant anaemia of chronic disease (ACD) secondary to infectious, neoplastic, or inflammatory diseases [12–15]. ACD is the consequence of the production of pro-inflammatory cytokines (IL1, 6, TNF*α*) [15] and some anti-inflammatory cytokines (IL-10) which induce the reticuloendothelial system to store Fe limiting its availability for erythropoiesis, decrease the half-life of erythrocytes, inhibit the production of erythropoietin (Epo) and decrease the sensitivity of erythroid precursors to Epo [16, 17]. Thus, ACD by itself results in hypoferremia and hyperferritinemia thereby complicating etiological diagnosis of patients with simultaneous IDA. Moreover, normal physiological levels of serum iron are difficult to establish in a population due to its circadian rhythm [18], technical limitations of the method, and frequent indication of ferrous salts [19].

In Fe deficiency, the decreased serum iron concentration leads to an increase in total capacity of iron binding (TIBC) and a decreased saturation of the iron transporter transferrin (Tsat). Ferritin (Ferr) and transferrin (Tf) have the disadvantage of being acute phase reactants with limited value in the differential diagnosis of ACD from IDA [20].

The above considerations justify efforts to design a highly sensitive and specific test to detect iron deficiency, ideally before the development of anaemia [21, 22]. Staining of the iron deposits in bone marrow (BM) remains as the gold standard, but it is an invasive technique. We assessed the use of soluble transferrin receptor (sTfR) [23, 24], present in the serum that can be easily quantified by conventional techniques and presents great potential for the distinction between IDA and ACD [24–26] highly necessary for the therapeutic treatment in the elderly. Its' concentration rises when there is marked lack of intracellular iron as the cell increases the number of receptors on its membrane [27, 28]. These parameters can eliminate the need of using BM aspirate to diagnose iron deficiency in some cases. However, it must be remembered that the sTfR commonly is ubiquitously expressed at low levels. Its expression can be elevated, in a variety of human cancers [29]. In addition to its role in iron metabolism it has been suggested that sTfR may play a role in cellular signaling and proliferation stimuli [30].

Our aim was to determine the prevalence of anaemia in a hospitalized elderly population and quantify the proportion that corresponds to iron deficiency using the ratio sTfR/log Ferr as a gold standard diagnostic parameter, as other authors [21], in order to assess later its utility in the differential diagnosis of ACD with IDA in comparison to other ferrokinetic and hematological classical parameters for use in the future in elderly patients.

## 2. Patients and Methods

We studied all the patients who were admitted at the Internal Medicine Ward at HV, Chile, between October 31 and December 31, 2008. Prior to informed consent, they were asked to participate in this research protocol without compromising medical care for their condition. Identification data and sociodemographic variables were asked (age, sex, marital status, health insurance, urban or rural residence, and educational level), and the cause of their hospitalization was obtained from the medical history.

We defined as elderly adults those with age ≥60 years. We assessed the presence of anaemia through the first inpatient complete blood count (CBC) using the World Health Organization definition: Hemoglobin (Hb) <13 g/dL for males and 12 g/dL for women. Anaemia was considered microcytic when MCV was ≤80 fl, macrocytic ≥100 fl, and normocytic 81–99 fl. The severity of anaemia was considered to be severe: (Hb < 7 g/dL), moderate (Hb 7–9 g/dL) and mild (Hb 9–11 g/dL). Patients with anaemia were further for analyzed serum iron, serum ferritin, TIBC, Tsat and sTfR quantification. The techniques used in the study and their normal values according to the manufacturer are shown in Table 1.

The ratio sTfR/logFerr was calculated in patients with ferritin <30 ng/mL considering the value <1 as compatible with ACD, >2 as ACD associated with another etiology using the flowchart of Weiss and Goodnough [23]. Statistical analysis was expressed as mean ± SD or range of distribution if not distributed normally. Statistical calculations were performed with Epidat 3.1.

## 3. Results

During the study period, 391 patients were hospitalized, out of which 247 were over 60 years. Details of their sociodemographic variables are shown in Table 2. It was not possible to survey 44 patients because of their clinical features (confusion, dementia, etc.).

**Figure 1:**
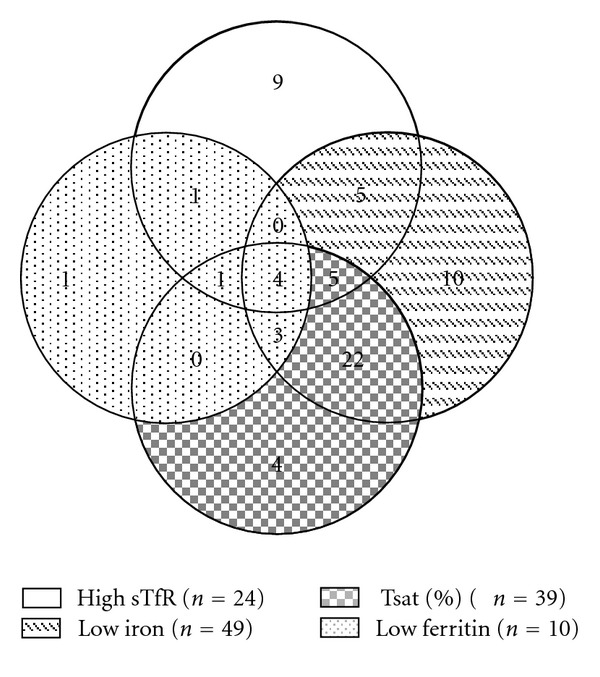
Schematic representation of the number of patients in each category according to their ferrokinetic tests results.

The patients hospitalized (*n* = 391) had an average hospitalization time of 6.3 days, with an average age of 62.5 years (15–95 years) of which a 63.15% were elderly. The CBC assessment revealed 99 elderly patients with anaemia according to the WHO criteria. This allowed us to calculate a prevalence of anaemia of 40.1% in the elderly inpatient population. In this population, the mean age was 73.2 years (60–90); the distribution by sex was 59.6% female and 40.4% male. The first three causes of hospitalization were in order of frequency: acute lung infection 12.5%, heart failure 10.2% and acute coronary syndrome with a 9.9%. Analysis of the haematological parameters revealed an anaemia distribution according to morphology of 88.8% normocytic, 9.1% microcytic and 2% macrocytic; and according to severity: severe 3% (Hb < 7 g/dL), moderate 25% (Hb 7–9 g/dL), mild 39% (Hb 9–11 g/dL), and of lower intensity 32% (Hb 11 to 12.9 g/dL). Ferrokinetic analysis revealed (see Figure 1) that serum iron was low in 50.5%, normal in 47.5%, and high in 2%; ferritin was low in 10.1%, normal in 52.5%, and high in 37.4%; TIBC was low in 73.5%, normal in 23.5%, and high in 2%, Tsat was low in 40.2%, normal in 44.8%, and high in 14.6%, and the sTfR quantification was low in 1%, normal in 74.3%, and high in 24.7%. Figure 1, illustrate the variability of the analyzes ferrokinetics present in the same group of patients (Elderly anaemic). Example: Serum iron was low in 50.5% (*n* = 49 patients) and 14 of them had high sTfR. Ferritin was low in 10.1% (*n* = 10) and 6 of them had high sTfR, %Tsat was low in 40.2% (*n* = 39) and 10 of them had high sTfR.

**Figure 2:**
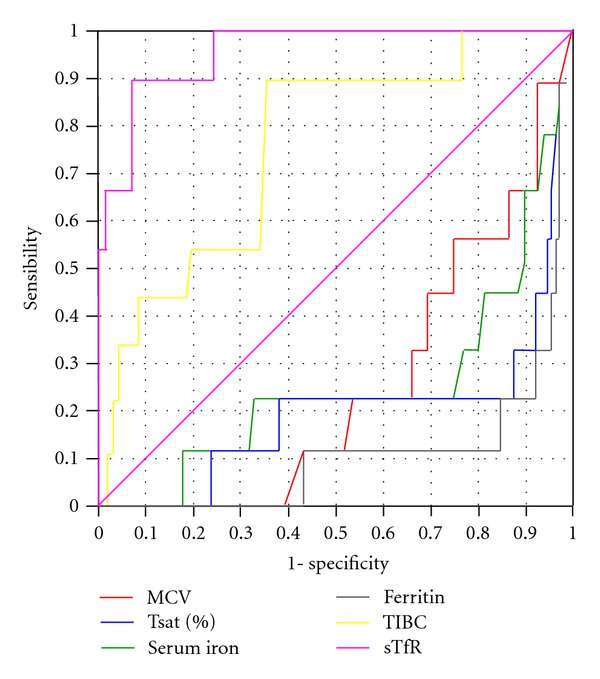
Receiver operating characteristic (ROC) curves for Serum iron, TIBC, Ferritin, % Tsat and sTfR for elderly patients with anaemia. sTfR/log Ferr ratio was considered the gold standard.

According to sTfR levels, patients were grouped into low 1%, normal 74%, and high 25%. Using the ferritin and sTfR/log Ferr values according to the algorithm suggested by Weiss [19], we concluded that there were 67% of ACD, 13.5% of IDA, 9.3% a combination of both, while 1.2% could not be categorized. The statistical analysis comparing the sensitivity and specificity of the sTfR/LogFerr ratio versus the other ferrokinetic parameters (Serum iron, TIBC, Tsat, ferritin, sTfR) was obtained from receiver operating characteristic (ROC) curves (Figure 2). The following areas under the curve were obtained for serum iron 0.2487, Tsat 0.1938 and ferritin 0.1061. sTfR and TIBC areas under curve was better: 0.9609; 0.7538, respectively. But when comparing the areas under the curve of sTfR and TIBC, any of them is a better diagnostic support if we do not have other test (e.g.,: Perl's stain), since both have asymptotic significance less than 0.05. However, the confidence interval for TIBC test was significantly lower than sTfR, which was a statistically significant difference between the two areas under described under the ROC curves (*χ* ^2^ test of homogeneity *P* = 0.0214). 

## 4. Conclusions

From an epidemiological point of view, the prevalence of anaemia was 40.1% for elderly hospitalized patients which is higher than that reported in other age groups in Chile and was unknown until now [6, 31, 32].

Our study confirms that sTfR quantification is a valid method to analyse the erythropoiesis in several diseases. To evaluate the clinical usefulness of sTfR in elderly patients for determining their iron deficiency status, receiver operating characteristic (ROC) curves were used, and the maximum discrimination cut-off point was calculated (see Figure 2). The sTfR was highly superior (sTfR area under ROC curve = 0.9609) to discriminate IDA from ACD in an adult patient population as compared to the classical ferrokinetics and haematological parameters. Ferrokinetic studies in the analyzed population were not useful for evaluating iron reserves and even were misleading in the diagnosis. A low ferritin concentration had been described as a good parameter for diagnosing iron deficiency anaemia [33] however, it only managed to predict 10% of patients with anaemia of this type in our study. The low Tsat was consistent with iron deficiency anaemia in 25%, but did not discriminate with ACD. This is consistent with the fact that transferrin, similar to ferritin, is an acute phase reactant which is elevated in inflammatory disorders [34], still both are widely used [19] in clinical practice due to the simpler technique and low cost. Furthermore, low levels of transferrin or TIBC may be due to increased degradation rather than decreased synthesis, as a result of increased protein catabolism secondary to catabolic or antianabolic proinflammatory cytokines [35, 36]. Our study confirms that the analysis of different individual ferrokinetic parameters is of little use in patients with concomitant systemic diseases, which are especially prevalent in the elderly; see ROC curves and areas.

In elderly adults requiring hospitalization, ACD was the primary cause of anaemia constituting over 75% of cases, while only 10% were due to iron deficiency. Folic acid and/or vitamin B12 deficiencies were not analyzed because it was beyond the scope of this study. Finally, the method of excellence to evaluate iron deposits remains bone marrow aspirate analysis with Perls stain constituting in some cases the only technique for diagnosing iron deficiency, however, this test cannot be performed routinely because it is invasive, expensive and slow [37, 38]. Our work shows that quantification of sTfR is more sensitive and specific for discriminating iron deficiency anaemia from anaemia associated with inflammatory events, consistent with work from others authors [21, 23, 39, 40], and will be a useful diagnostic tool for the future in our elderly patients. It is also necessary to note that most of the study population was admitted to the hospital primarily for cardiac diseases (29.22%), respiratory diseases (13.14%), and renal failure (6.17%). The latter is directly related to the generation of anemia by a low secretion of Epo, which must be considered when making the sTfR test if a patient with impaired renal function.

This sTfR/logFerr ratio gives us an inverse linear relationship of the iron stores state and has the advantage of combining the increase of one parameter (sTfR) and the decline of another (ferritin) [21, 41]. Our findings encourage us to promote the implementation of this determination (sTfR) in the clinical setting as was previously proposed [24, 26, 42], and high interest for the elderly adults. It is important to note that this method still requires international standardization in order to define normal ranges, which at the present time limits its routine clinical application [43].

## Figures and Tables

**Table 1:** Techniques for ferrokinetics and haematological parameters and normal values according to manufacturer and iron deficiency criteria used in the study.

	Method	Manufacturer‘s normal value	Iron deficiency criteria
Serum iron	Iron liquicolor photometric colorimetric test for iron with lipid clearing	Men 59–148 ug/dL Women 37–145 ug/dL	Men < 59 ug/dL Women < 37 ug/dL

Total iron-binding capacity (TIBC)	TIBC test, iron saturation and aluminum oxide absorption method, human	250–370 ug/dL	>370 ug/dL

Ferritin (Ferr)	Electrochemiluminescence immunoassay. Roche, Elecsys 2010/modular analytics	30–300 ng/mL	<30 ng/mL

% Transferrin saturation (% Tsat)	TIBC test/iron liquicolor, human	20%–45% ref	<20%

Soluble transferrin receptor (sTfR)	Enzyme-linked quantikine IVD human sTfR immunoassay	8.7–28.1 nmol/L	>28,1 nmol/L

Ratio sTfR/log Ferritin (sTfR/log Ferr)		1-2	>2

MCV	SYSMEX. XE-alphaN automated hematology system roche,	80–99 fL	≤80 fL

CHCM	SYSMEX. XE-alphaN, automated hematology system roche,	32–36 ug/dL	≤32 ug/dL

**Table 2:** Main demographic variables of hospitalized patients, elderly and those elderly with anaemia.

	Total patients admitted	Elderly patients	Elderly patients with anaemia
Number of cases	391	247	99
Mean Age (years)	62.5	73	73.2
Residence			
Urban	280	180	65
Rural	95	60	27
Not available	16	7	6
Literacy			
Yes	288	161	46
No	47	41	20
Not available	56	45	33
Gender(%)			
Male	192	118	40
Female	199	129	59
